# Qualitative Study on the Toxic Triangle Integration of Leadership Ostracism

**DOI:** 10.3389/fpsyg.2021.655216

**Published:** 2021-07-29

**Authors:** Zhixia Chen, Mei Sun

**Affiliations:** Department of Public Administration, College of Public Administration, Huazhong University of Science and Technology, Wuhan, China

**Keywords:** leadership ostracism, power distance, political skills, Chaxu climate, toxic triangle model

## Abstract

Leadership ostracism widely exists in all types of organizations, yet specific study regarding this trend is limited. With this study, we explore the influencing mechanisms of leadership ostracism through case interview based on literature analysis and grounded theory. Results show that leadership ostracism is the integration of a triadic interaction process between subordinate performance, leadership characteristics, and organizational environment. Based on Padilla's destructive leadership toxic triangle model, we constructed a toxic triangle model of leadership ostracism. Through comparison, we found that these two triad models overlap in the areas of narcissism and power consciousness of supervisors, the self-concept of subordinates, and the management system of situational factors, indicating that leadership ostracism is itself a type of destructive leadership. In addition, the uniqueness, and differences in leadership ostracism are reflected in the model, including stereotypes, and results orientation of supervisors, political skills, job performance, and cognitive style of subordinates, the power distance, *Chaxu* climate, and organizational politics of the situational elements. Theoretical and practical implications are discussed in the research field that provides prospects for future orientation.

## Introduction

Workplace ostracism refers to “the extent to which an individual perceives that he or she is ignored, rejected or excluded by others at work” (Ferris et al., 2008). Constructs for workplace ostracism are noted in collective experiences such as exclusion (Pereira et al., 2013), social rejection (Haldorai et al., 2020), organizational shunning (Quade et al., 2018), and feeling “out of the loop” (Robinson et al., 2013). Even in subtle forms, an ostracized individual may respond with physical chills, and psychological reactions, such as nervousness, and sadness (Howard et al., 2019). To elucidate the potential sources and perpetrators of ostracism, Ferris et al. (2008) differentiate workplace ostracism into (1) leadership ostracism and (2) coworker ostracism. Leadership ostracism is a common phenomenon and primarily manifests as a form of authority exerting influence on promotions, rewards, and resource acquisition of subordinates; thus, its negative effects, and consequences are more serious than that of co-worker ostracism (Hitlan and Noel, 2009). Leadership ostracism may lead to an increase in deviant, unethical, and/or counterproductive behaviors resulting in negative consequences on job performance and organizational outcomes (Martinko et al., 2013; Chung, 2017; Sarwar et al., 2020). Given its effect, an increase in research on leadership ostracism has emerged exposing the dark side of negative leadership (Jahanzeb et al., 2018; Kanwal et al., 2019; Zhao et al., 2019). Less clear, however, is when and why supervisors engage in leadership ostracism.

Understanding the antecedents of leadership ostracism is crucial; however, several antecedent factors have merged in the accumulated findings over the past decade (Ferris et al., 2015). The predominant studies in leadership ostracism literature have focused on certain subsets of personality or supervisor–subordinate relationships while offering little insight into establishing a holistic antecedent framework (Hitlan and Noel, 2009; Xu et al., 2015). For example, models to explore how a victim may trigger a situation where they are ostracized by a supervisor are built around the individual characteristics of the subordinate from the perspective of behavioral recipients (Xue et al., 2020). Another stream of study on the perpetrators explores the intention of supervisors to ostracize and leadership styles under contextual stress (Schyns and Schilling, 2013; Quade et al., 2018). In this paper, we follow a holistic perspective and construct a theoretical framework that integrates the victim, the perpetrator, and the contextual factors.

Given that there is no established framework available on leadership ostracism, a qualitative research method is more suitable to explore the antecedents (Wilhelmy et al., 2016). First, through literature review, we conceptually define leadership ostracism and clarify what type of situation characterizes leadership ostracism for the victim. Next, by conducting interviews, we analyze practical cases collected in a field setting in which leadership ostracism occurred to identify key antecedents. Consistent with Padilla's toxic triangle model for destructive leadership, we then divided the results into factors (1) supervisor, (2) subordinate, and (3) organizational context. As a form of destructive leadership, leadership ostracism applies to “follower-targeted influence” that builds on leadership positions and contextual interactions (Schyns and Schilling, 2013). In the current study, we extended the toxic triangle model on the antecedents of leadership ostracism from the supervisor, subordinate, and organizational context, and we discuss the principal personal and environmental predictors in these three domains. By testing a dynamic conceptual model of leadership ostracism, this study makes contributions to the leadership literature and offers theoretical and practical implications for improving organizational climate and employee well-being.

## Theoretical Backgrounds

**Figure 1:**
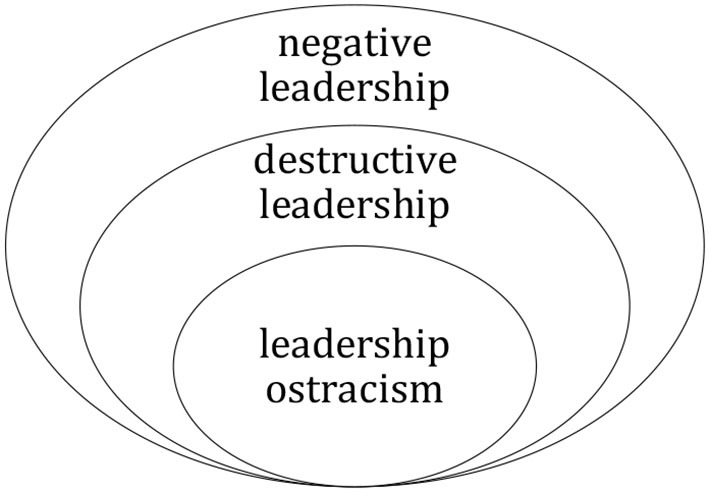
Venn diagram of negative leadership, destructive leadership, and leadership ostracism.

Leadership (supervision) ostracism is a subtle yet universal form of interpersonal mistreatment that employees perceive from supervisors, which includes neglect, rejection, and exclusion (Ferris et al., 2008; Chen and Tu, 2017). From a victim-focused viewpoint, this mistreatment is a subjective experience and the perception of subordinates that suffer ostracism by someone in a position of authority. Affected by authority hierarchy, subordinates are “more astute at identifying ostracism from superiors” than other types of workplace ostracism, which can be attributed to their need for leadership support, approval, and advancement in job evaluation (Zhao et al., 2019). Leadership ostracism includes both behavioral actions (e.g., excluding, rejecting), and relatively static inaction (e.g., ignoring, neglecting, shunning). However, leadership ostracism is principally more of a silence/emotional intimidation in the workplace that does not include either direct verbal or behavioral conflicts. Even though leadership ostracism is generally a low-intensity conflict, it frequently occurs in conjunction with other forms of destructive leadership, including incivility, tyranny, bullying, abusive supervision, and/or physical assault. Therefore, it is crucial that leadership ostracism be included in discussions on leadership. In terms of concept, connotation, and performance, leadership ostracism is included in destructive leadership, and destructive leadership is included in negative leadership (see Figure 1).

The current study differentiates leadership ostracism by three aspects. First, behavioral motivation–leadership ostracism may be a strategy utilized to obtain higher job performance and increased organizational outcomes whereby supervisors exert a “bad” influence on subordinates whether the employee performs well or not (Vidyarthi et al., 2014; Howard et al., 2019). Other types of destructive leadership, such as abusive supervision, may result from metamorphosis or dark personality of a supervisor and frequently target high-performance employees (Tepper, 2007). Next, target influence differs in the workplace. Leadership ostracism is a type of silent intimidation manifest in leadership authority. Other destructive leadership behaviors commonly include a form of intense verbal or behavioral conflict, including negative behaviors directed at both subordinates (e.g., abuse, punching, and sexual assault) and the organization (e.g., deception, theft, and corruption) (Einarsen et al., 2007). Lastly, the scope and frequency of occurrence confine to different boundaries. Leadership ostracism is a common phenomenon. Interpersonal relationships form kinds of *Quanzi* that indicate the closeness of a subordinate–supervisor relationship, thus forming the organizational *Chaxu* climate (Chen and Dian, 2018). This elicits varied treatment of employees, while some subordinates in the *Quanzi* are always treated with preference. However, other destructive leadership behaviors occur only in certain circumstances, for example, when a leader is feeling depleted or when subordinates are considered a burden (Byrne et al., 2014; Ferris et al., 2015).

We adapted Padilla's toxic triangle framework to explore the antecedents of leadership ostracism. The framework includes “a confluence of leaders, followers, and environmental factors” (Padilla et al., 2007) that associate with personnel and contextual characteristics to fuel leadership ostracism. However, what leads to destructive leadership in the existing studies is limited to literature review; therefore, we discern a clear call for further empirical evidence. In the current study, we attempt to conduct a “new” toxic triangle model on the integrations of leadership ostracism by conducting in-depth interviews.

## Methodology

### Sample and Data Collection

Identifying and establishing antecedent frameworks for leadership ostracism is a somewhat new endeavor; therefore, we have taken a qualitative approach by conducting individual in-depth interviews from practical cases. Inductive case analysis is more intuitive, dynamic, and comprehensive (Eisenhardt and Graebner, 2007), and interview data can provide further insights into theoretical depth and breadth (Kelley et al., 2003). In the current study, a semi-structured interview was conducted to unveil when and why supervisors engage in leadership ostracism in the workplace.

Before the study began, we generated a list of 87 Master of Public Administration (MPA) students from various professions who, based on daily contact, and knowledge, reported situations of distress, and rejection by their supervisors or had observed colleagues being ostracized. According to theoretical sampling methods in grounded theory, 20–40 participants would meet recommended sample sizes (Hays et al., 2016); we, therefore, randomly selected 40 of the 87 participants and nine respondents declined. Next, we eliminated interference factors by applying selection criteria as follows: full-time, regular employee who had worked for more than half a year; recruitment, not appointment from the top; had frequent opportunities to work directly with leaders; undertook substantive work within the organization or held a specific position; not the subordinate of only leaders. During this stage, we excluded five individuals from our sample and an additional three individuals chose to discontinue due to personal reasons. Finally, we took 23 respondents as final samples based on the rule of “theoretical saturation” to assure that key points were common and not just “random occurrences” (Waldeck et al., 2015; Heyler et al., 2016).

**Figure 2:**
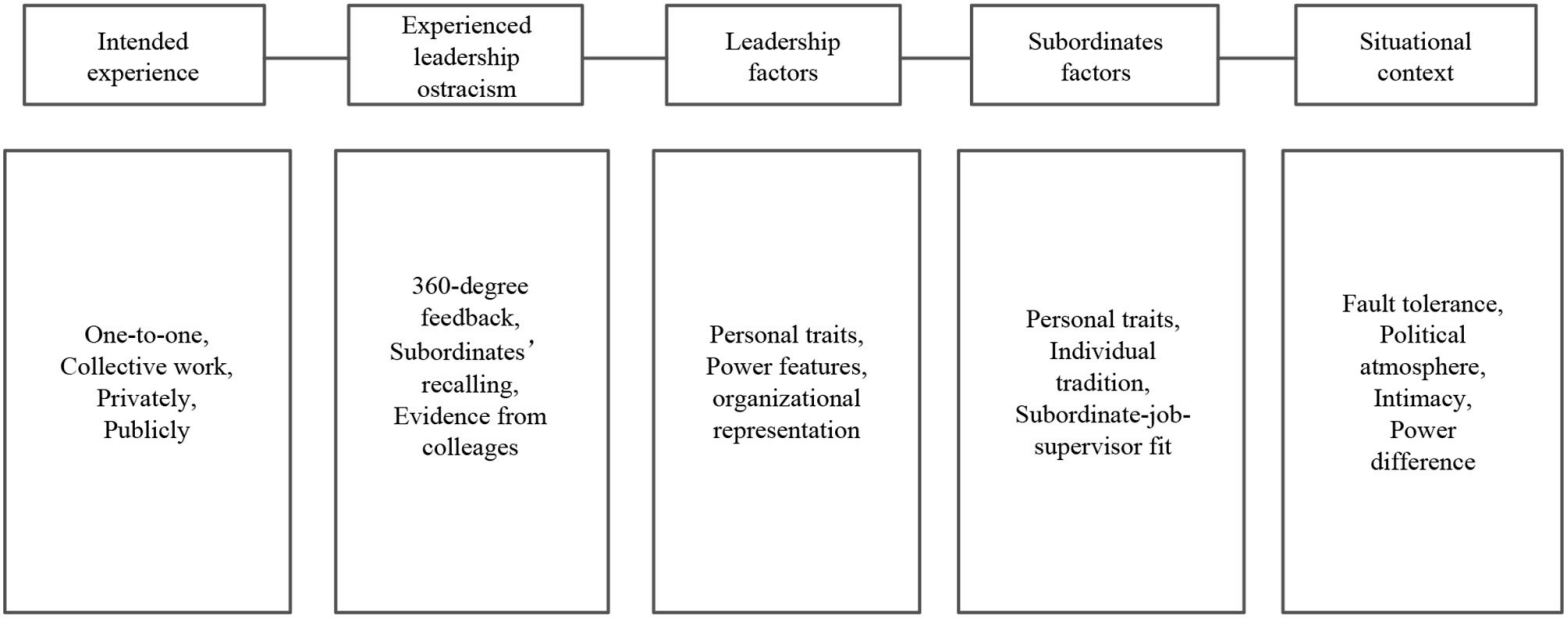
Guiding investigation framework.

The interview guide is shown in Figure 2. *A priori* framework specification for research questions served as a base for theory-building in the case study (Eisenhardt, 1989). We contrasted the assumed and experienced practices from an individual-level perspective to identify the characteristics of leadership ostracism and examined how individuals became victims and experienced leadership ostracism. Taking Company A as an example, since the staff in the department had established long-term contact with our project, we were permitted to carry out preliminary testing on leadership ostracism. In our research, a framework that connected “post-positivist, constructionist, and interpretivist approaches” of grounded theory was applied to explore how leadership ostracism was formed (Levers, 2013). On the one hand, we attempted to combine existing theoretical constructs with intuitive ideas of individuals on leadership ostracism to achieve a more accurate prediction of behavior (Webster et al., 2008). In addition to the literature review, we conducted one-on-one interviews to understand what was perceived as leadership ostracism, we checked victim resource allocation and task assignment by reviewing work documents in collective work, and we personally observed the treatment of victims by their leaders in private and in public settings by participating in organizational programs. On the other hand, we learned about ostracism practices in the workplace through informal conversations with leaders, subordinates, and colleagues, whose feedback was primarily based on firsthand experience or daily observations. These data would complement our previous observations and formed the overall description of leadership ostracism and its antecedents.

The final research sample includes the following statistics: Gender = 10 males/13 females; Age of participants = 16 (25–30 years), 4 (31–40 years), 2 (under 25 years), and 1 (40+ years); Organization type=9 from public sector, three from state-owned sector, and 11 from private sector; Working experience = 87% with 1–5 years, and Work type = 91.3% are office juniors and mid-level office staff. Due to restrictions related to the working hours and job requirements of participants, the interviews were conducted by either one-on-one field interviews, telephone calls, or an online survey.

To create a relaxed conversational atmosphere, we began with light topics such as career development plans and expectations for ideal leaders. Then, we conducted interviews following the investigation framework (see Figure 2) in a semi-structured manner by a chief investigator and two assistants, which lasted 60 to 90 mi using audio and handwriting records. Respondents recalled critical incidents on leadership ostracism and contrasted themselves with other colleagues. Appendix A contains nine items on leadership ostracism gathered from the interviews.

### Ethical Statement

Ethical approval was not required because this research was conducted in accordance with institutional regulations and did not involve clinical or animal trials. Furthermore, in accordance with the Declaration of Helsinki, throughout the process we followed ethical guidelines and obtained written consent after participants were informed of the research purpose. Participants were informed that the experience of recalling leadership ostracism might be psychologically uncomfortable. They could terminate their participation in the study and discontinue at any time if they choose. Our research was anonymous and confidential.

### Data Analysis

Based on grounded theory, we conducted inductive analysis and explored the inherent logic from practical cases. It included encoding and categorization in a continual process to identify concepts, eliminate interference, and develop a new theoretical framework (Katz, 2015). In the first step, two Ph.D. students independently conducted a preliminary content analysis by screening, sorting, labeling, and coding the interview data. Next, they held an in-person discussion regarding the challenges and differences and conducted a comparative analysis on mutual coding results until a consensus was reached (Wilhelmy et al., 2016). To ensure objectivity, original phrases or descriptions that reflected the same behavior were kept and labeled as the first-order code (Sbaraini et al., 2011). By conceptualization, synonymous expressions that had similar meaning were integrated and renamed. For example, “judging people by appearance” and “fond of handsome subordinate” were considered as synonymous expressions and could be conceptualized under the label “appearance stereotype.” In this process, we were also concerned with the relevance of our preliminary concepts and existing items from interpretivist perspective and constructivist perspective, because our intention is to expand on current theoretical framework (Shah and Corley, 2006; Levers, 2013).

The second step was axial coding that aimed to identify core categories and dimensions by relating independent concepts. A coding paradigm that included contextual conditions, subject interactions, and behavioral consequence was applied to explore the relationship between concepts and then form different levels of categories (Suddaby, 2006). Through repeated intra-group and inter-group comparisons, we tried to eliminate differences on conceptual classification and seek consensus. Additionally, we provided “member check”–feedback on the categorized classifications to participants who could choose items based on individual experience and gave them opportunities to express opinions (Stivers, 2007). Finally, selective coding was conducted to systematically integrate the relationships between key categories. The coding results indicate that the antecedents of leadership ostracism focus on the behavioral subjects–supervisors (perpetrators) and subordinates (victims), and organizational context.

## Results: How is Leadership Ostracism Formed?

**Figure 3:**
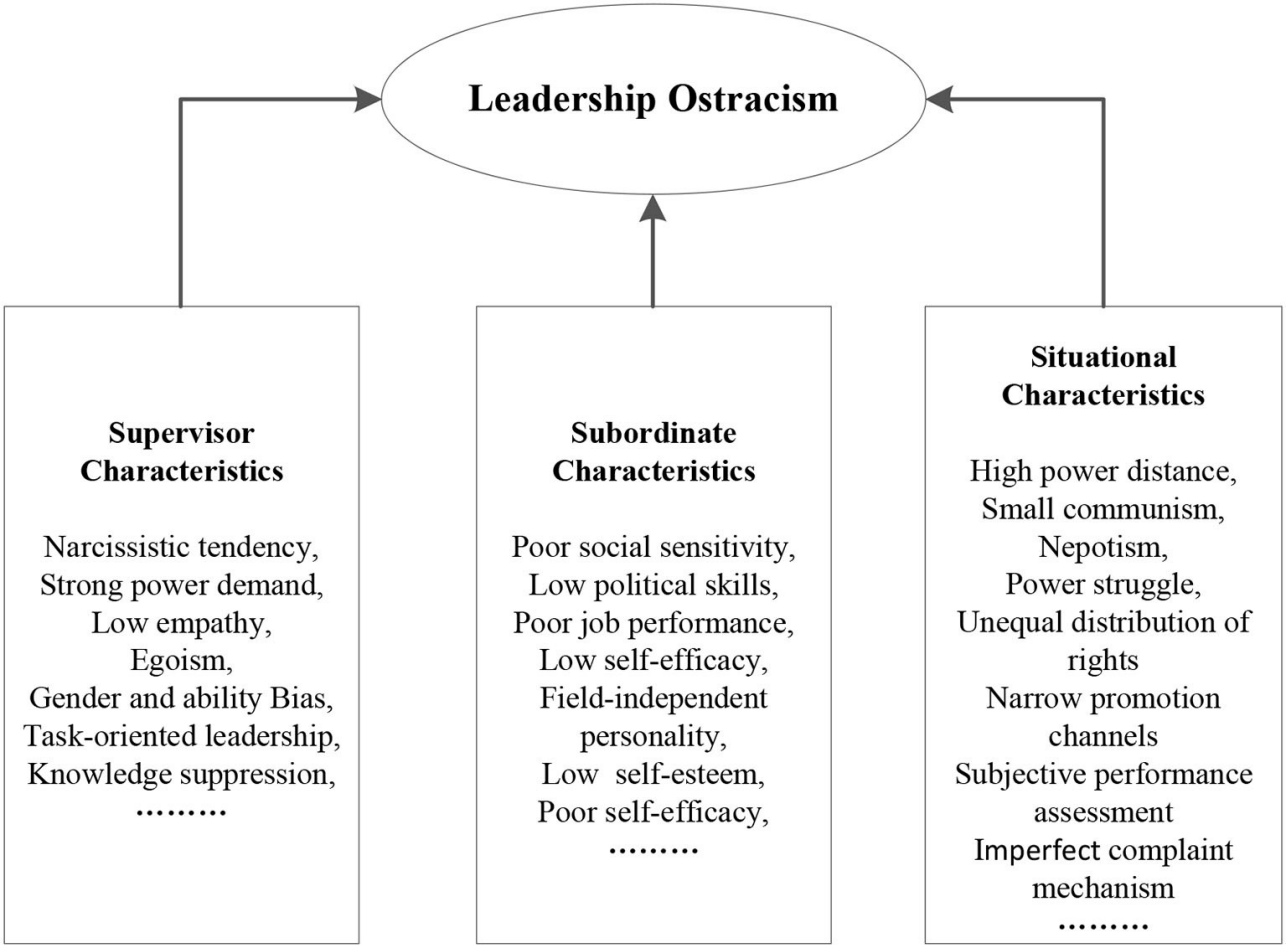
Inductive model of Leadership ostracism.

Leadership ostracism is a widespread phenomenon in the workplace (Jahanzeb et al., 2018). It is a simultaneous effect of supervisor, subordinate, and organizational context (as shown in Figure 3). In the coding process, we identity three key categories, 13 main categories, 29 subcategories from more than 129 initial phrases (see Table 1). Specially, the core concepts include *supervisor traits* (involving narcissistic tendencies, power awareness, stereotype and prejudice, result orientation, and unfairness), *subordinate traits* (involving political skills, personality, self-concept, and job performance), and *organizational context* (involving power distance, Chaxu climate, organizational culture, promotion channels, and complaint and appeal mechanisms).

**Table 1:** Results of interviews.

**Key categories**	**Main categories**	**Corresponding categories**	**Initial concept**
Leader/supervisor	Personality	Narcissistic	Self-orientation, face-saving, enjoying ingratiation, praise addiction, arrogant, self-righteous……
		Narrow-minded	Narrow-minded, intolerant to different voice, strong jealousy, afraid others to exceed themselves……
		Selfish	Avenging a personal wrong in the name of public interests, using public power for the private purpose, doing everything guided by their own interests……
		Lack of empathy	Only thinking things in one's own perspective, unable to stand in others' shoes and set oneself up for others' sake……
		Low EQ	Poor emotional adjustment ability and poor interpersonal coordination……
		Dark personality	Machiavelli doctrine, neuroticism, psychosis, and narcissism, selfishness, irresponsibility, and relentlessness, bad temper……
	Power tendency	Keen on power	Strong power desire, strong concept of hierarchy, high power distance leader, strong traditional thinking mode, one person lays down the law, not taking the initiative to approach the subordinates, bureaucratic style, wanton, and standing upon one's pantiles……
		Jealous and envious	Selfish, low sense of psychological security, worrying of surpass by their subordinates, taking success as himself, and underling subordinates……
	Stereotype and Prejudice	Appearance stereotype	Judging people by appearance, fond of beautiful and charming young girls, fond of handsome subordinate
		Gender stereotype	Looking down on and despising women, believing female employees are inferior in ability and poorer in career……
		Age and education stereotype ……	Disliking elderly employees, disliking employees with low degrees of education……
	Result orientation	Task-centered	Only caring about tasks, showing no concern about people, interest orientation of quick profit and instant benefit, lack of humanistic care……
		Machiavellianism	fond of political trickery, believing the end justifies means
	Unfairness	Leadership unfairness	Lack of self-cultivation and equality consciousness, unfair treatment of subordinates, their own preferences are always the primary concern, believing that those who resist shall perish……
Subordinate	Political skill	Ingratiation	Poor at dealing with interpersonal relationship, insensitive to other's language and countenance, disliking making up to the boss, unable to understand the supervisors' preferences, paying no attention to the cultivation of the same hobby with the leader, and choosing unwisely……
		Low EQ	Clumsy words, not actively communicating with supervisor, making supervisor feel embarrassed, disregard for the occasion, fond of challenging the authority of the supervisor, to marginalization of the subordinate themselves……
	Personality	Independent	Field independence type of personality, too upright, unruly, unfriendly, uncooperative, not in line with leadership's work, and even staging a rival show with supervisor……
		Dark personality	Machiavelli doctrine, neuroticism, psychosis and narcissism, selfishness, and relentlessness……
	Self-concept	Low moral traits	Lower job ability, lower practical skill, simple ability structure, low job performance, poor interpersonal relationships, negative job attitudes, and no desire to make progress, bad habits or problem behaviors, Machiavellianism, selfish, mean, petty, cold, rude, incivility, seeking nothing but profit, low team spirit………
	Job performance	Unmatched with supervisor	Whose appearance, behavior, personality, hobbies, values and so on are not leaders' favorite types……
		Opposite to supervisor	High performance but independent, threat to a supervisor, informer leadership, opposite to supervisor, high prestige in informer group……
Organizational environment	Power distance	The sense of hierarchy	Strong sense of hierarchy, subordinates must submit themselves to absolute obedience……
		Supervision power	Overpower of leadership, do whatever he wants, like who then who is………
	Chaxu climate	Guanxi orientation	Guanxi-oriented rather than goal-oriented, task-oriented and performance-oriented, lack of organizational fairness atmosphere
		Circle Atmosphere	The emergence and existence of organizational factions, insiders and outsiders, small groups……
	Organizational culture	Organizational cohesion	Low organizational cohesion, low organizational sense of identity and low organizational commitment……
		Competition relationship	Wolf culture emphasis of competition or fierce competition, lack of cooperation spirit……
		Climate	Colleagues keeping a safe watch, following the crowd, fighting with each other, making small reports, gossip, repelling, dismantling each other, or even dropping stones on the man who has fallen into a well……
	Management system	Management system	Problems on employee complaints and rights protection system, employees are put on a relatively weak side in the organization with heavy psychological pain……

### Supervisor Factors

#### Narcissistic Tendencies

During the interview process, participants described their leaders as narcissistic valuing only the achievement of their own personal goals or self-interests. They reported a tendency toward partiality to subordinates who are obedient, flatter them, and are good at catering to their emotions and preferences while displaying a hostile and negative attitude toward subordinate feedback. For example, sample reports were as follows:

“*In public, my boss attached great importance to maintaining his image and authority. He was very sensitive to criticism and desired for praise.”*

“*My supervisor was narcissistic. He paid much attention on interpersonal intimacy and was keen to establish small groups in the organization.”*

#### Power Awareness

When public and private rights overlap, leadership ostracism is easily facilitated (Vidyarthi et al., 2014). Participants reported that their leaders had a heightened power awareness and ostracized employees who displayed excellent abilities. Additionally, they treated competent subordinates as potential threats and regarded interpersonal interactions as a “zero-sum game.” They might reject subordinates with outstanding ability, deprived them of opportunities to perform, or embarrassed them publicly. For example, sample reports were as follows:

“*My boss was arbitrarily authoritative and suspicious. He had a strong sense of power and disliked subordinates who displayed excellent abilities.”*

“*My supervisor demanded absolute obedience to his orders. He issued direct commands in our daily work and did not like to be opposed.”*

“*My leader only concerned with work efficiency and outcomes in practice, lacking care and help for us.”*

#### Stereotypes

Stereotypes are fixed ideas or opinions toward certain social members (Purdie-Vaughns et al., 2008). Individuals are often judged, regarded, treated, and restricted to their social identity in given settings (Hoyt and Murphy, 2016). Noted in the interview for subordinates was that they were prone to suffering prejudice manifested as gender bias, ability bias, and age bias. An example was that female employees were considered good at “take care” responsibilities rather than “take charge” opportunities and were therefore overlooked for recognition or promotion (Hoyt, 2010). Other sample descriptions were as follows:

“*The supervisor valued work ability and excluded the subordinates who could not meet the desired goals.”*

“*She had an obvious gender bias. She treated women subordinates badly and refused to assign them important tasks.”*

“*My new leader never allowed the old staff to attend important meetings, nor arranged them important work.”*

#### Results Orientation

Respondents reported that their leaders had a strong sense of achievement and low emotional empathy, and they eagerly pursued work efficiency and committed to promotion. They also reported that their supervisors lacked a compassionate humanistic concern for their subordinates. For this type of supervisor, work results are the only basis for evaluations. When a subordinate has a poor work ability or may not attain expected goals, they are regarded as a burden and consequently become the target of leadership ostracism behaviors.

“*My supervisor was obsessed with high job performance. If you did not meet his requirements, he would think you were incompetent and handed over relevant tasks to other colleagues.”*

“*Given that Li had failed several times to complete urgent tasks on schedule, his boss transferred him to the sales department by job rotation. He was unable to adapt to new job and considered quitting.”*

### Subordinate Factors

#### Political Skills

In the coding process, we found that in the course of daily work, the ostracized employees lacked the ability to influence others toward realizing individual or organizational goals, which could be regarded as a challenge to leadership authority. They frequently disregarded organizational rules, preferences of leaders, and their own strengths/weaknesses, and they barely perceived environmental changes or made behavioral adjustments appropriate to the occasion. Sample statements were as follows:

“*Wang was reluctant to communicate with others and looked down on others who cater to leaders' preferences."*

“*He was a maverick and always rubbed against his boss.”*

“*I was a simple and independent person. I did not prefer to deal with my boss direct. Although I was reminded to cater to leaders' preferences, I would not do that.”*

#### Personality

Field-independent subordinates tend to have less dependence on external factors and are not as easily affected by the assimilation effect in the workplace. However, this may also be conducive to friction with supervisors. Respondents recalled that victims maintained moral ambitions and refused to conspire with the leaders, especially when they were instructed to violate ethics or damage organizational interests. Therefore, employees who were not inclined to proffering flattery or who pursued their own individuality were ostracized or became targets for exclusion by their supervisors.

“*She adhered to basic principles and professional ethics in her work, and always refused to seek personal benefits for her leaders.”*

“*Wang was simple, conscientious and independent. He refused to flatter his boss like other colleagues. Recently, he found that although he worked hard, the job evaluation was not satisfactory.”*

“*Zhang had a straightforward character. He clashed with his boss several times and made him embarrassed in public. His colleagues had already been promoted, but he remained in the same post for several years.”*

#### Self-Concept

Low self-concept subordinates lack self-esteem and self-efficacy and tend to have higher levels of neuroticism (Ferris et al., 2015). Thus, they are more easily affected by external context accompanied by low job ability. They have poor resistance to pressure and often fall into depression and stagnation once confronted with criticism and/or blame from supervisors.

“*She was timid and dull, and had poor job performance. She always denied his own ability and had a low self-evaluation.”*

“*The supervisor thought highly of him, but Li always took an arrogant attitude toward supervisors. He was perfunctory about the work assigned to him.”*

“*Tang was once publicly criticized by his boss for a technical mistake. He believed that his ability was not recognized. Gradually, his working attitude became more negative.”*

#### Job Performance

Supervisors tend to take punitive measures on subordinates who cannot achieve job objectives or adapt to high-intensity environments (Wesselmann et al., 2012), while employees who have high job performance may be perceived as potential threats by their supervisors. Respondents shared their experience as follows:

“*She had excellent ability to handle the job. However, the supervisor seemed to be hostile to her. He often deliberately made her embarrassed in public and arranged her some urgent and heavy works.”*

“*Zhou was a new employee who often volunteered to do some cleaning or printing work. However, her work performance was very poor. Her boss was dissatisfied and handed over her work tasks to other colleagues.”*

### Organizational Context Factors

#### Power Distance

In high-power distance organizations, members generally accept unequal power distribution and lack a spirit of cooperation (Vidyarthi et al., 2014). Subordinates with low-power distance orientation are vulnerable to leadership ostracism because they cannot meet the expectations of leaders.

“*There were complex relationships and hierarchies in our organization, and individual employees had to be careful to avoid being suppressed.”*

“*Promotion within our institute was based on the recommendations of our supervisor, and management system did not always work that was more of a formalism.”*

“*In my company, supervisors had absolute authority and dominated the career development of employees.”*

#### Chaxu Climate

In the Chinese context, awareness and *Chaxu* climate are widespread, which specify different interpersonal affinity relationships (Chen and Dian, 2018). Supervisors take interpersonal intimacy as a criterion for resource allocation and job evaluation. For employees who are out of *Quanzi*, they would have lower expectations toward supervisors, and to reduce the ostracism they experience, they may focus on how to transform themselves into “insiders.”

“*Those who had a good relationship with supervisors, even if their performance was poor, their job evaluations could always be excellent.”*

“*A close relationship with our boss was the only way to get training and/or promotion opportunities.”*

“*Our supervisor always gave priority to those who were close to him, rather than those who had excellent job skills and performance.”*

#### Organizational Culture

Interviewees reported that some organizational members who took unethical measures to obtain resources, achieve benefits, and serve self-needs would not be punished by the organization in general. The decision-making process, employment system, and compensation structure were not clear or transparent. Respondents perceived that rewards and punishments came from a position of power or a result of nepotism or the revenge mechanism; thus, they decided to change their career development strategy.

“*There were small groups in the organization, and individuals sought out the cliques that were best for them and actively joined them.”*

“*Chen was a member of the group led by the former leader, who was deeply disliked by the current leader.”*

“*The power struggle in our company was intense and it was important for us to choose factions.”*

#### Management System

Participants reported problems within the organizational management system. For example, regarding promotion opportunities, a supervisor might hinder the career development of a subordinate simply on personal preference. Performance evaluation criteria were subjective and came primarily from the opinions of the leader. Retaliation was allowed in the organization, and employees did not have rights of appeal or recourse. It is worth noting that a lack of supervisory control and organizational support would make perpetrators believe that their behaviors were recognized, warranted, or expected, and this contributes to sense of helplessness of victims (Balliet and Ferrsis, 2013).

“*Our boss had absolute authority over our promotion and performance evaluation.”*

“*It was hard for us to participate in making important decisions in our organization, and commands were issued from top-down that cannot be questioned.”*

“*I was afraid to tell the truth in the workplace, because it would surely lead to revenge from my boss.”*

“*We had no place to complain because there were few complaints and supervision channels in our institute.”*

## Conclusion and Discussion

Based on the grounded theory and multiple interview cases, we explore the antecedents of leadership ostracism and establish “a unifying theoretical framework” on extant research from three perspectives—supervisor, subordinate, and organizational context.

### Toward a Model of Toxic Triangle on the Antecedents of Leadership Ostracism

The concept of the toxic triangle was first proposed by Padilla et al. Based on literature review, they explored the origin of destructive leadership and took Fidel's personal career as an example. Padilla et al. (2007) believed that the emergence of destructive leadership was a dynamic combined effect of destructive leaders, susceptible followers, and conducive environments. Their proposed toxic triangle model of destructive leadership focuses on its “problematic or even disastrous outcomes” (Padilla et al., 2007). It breaks with conventional wisdom that destructive leadership comes from dark, abnormal traits, or behaviors of leaders but takes subordinates and organizational circumstances into account as well. Specifically, “destructive leaders” include characteristics such as personal charisma, power demands, narcissism, negative life experiences, and hate awareness. “Susceptible followers” include unquestioning obedience manifested by unmet needs and low maturity and/or collaborators who share similar worldviews, malevolent values, and personal ambitions with supervisors. Lastly, “conducive environment” includes instability, perceived threats, cultural values, checks and balances, and institutions.

**Figure 4:**
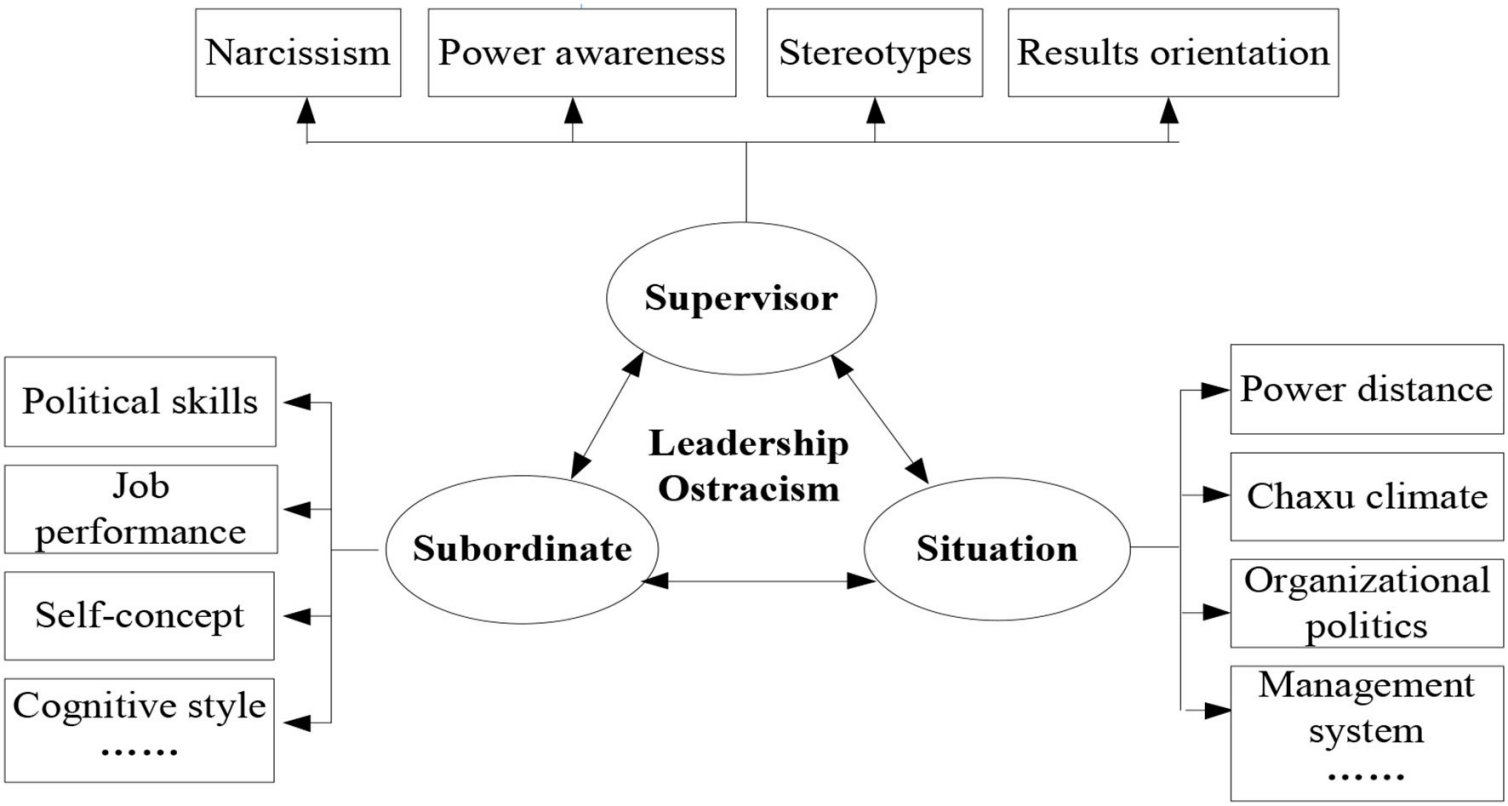
The toxic triangle model of leadership ostracism.

In this paper, we conducted an extension to the leadership research by qualitative method to explore the antecedents of leadership ostracism through case interviews. The case interview was conducted with participants who had encountered or observed other colleagues suffer with leadership ostracism. We take a multicase analysis on an item-by-item replication and differentiation logic to elucidate. Results indicate that leadership ostracism results from an integration of destructive supervisors, susceptible subordinates, and conducive organizational context. This is a “new” toxic triangle model of leadership ostracism based on the original theoretical framework (see Figure 4). To be specific, supervisor traits include narcissistic tendencies, power awareness, result orientation, and stereotypes and prejudices; subordinate factors include self-recognition, behavior style, job performance, and political skills; organizational context factors include power distance, Chaxu climate, promotion channels, and complaint and appeal mechanisms.

## Implications

### Theoretical Implications

There are several theoretical implications to our research. First, regarding the supervisor factors, we found that narcissistic tendencies, power awareness, stereotypes, and results orientation may trigger ostracism inclination of a supervisor. Although research has explored the traits and roles of ostracizers, such as power and status authority (Fiset et al., 2017), studies are relatively scant and there is not yet a unified research framework. Through case studies, this paper systematically explores the leadership factors that determine the extent to which supervisors practice leadership ostracism. Because leadership ostracism is a function of supervisors who act as perpetrators, so the emergence of supervisor characteristics can be regarded as a signal effect of leadership ostracism (Howard et al., 2019).

Second, regarding the subordinate factors, our study revealed that political skills, personality, self-concept, and job performance may make the mentally or physically vulnerable employees become ostracized targets. Extant research has presented the detrimental influence of leadership ostracism on personal performance with little attention on the reflections of subordinates themselves as the ostracized individual (Xu et al., 2015). Essentially, leadership ostracism is an interactive relationship between supervisors and subordinates; hence, it is not a single individual force that determines who suffers as a victim of leadership ostracism (Wan et al., 2016). Furthermore, another valuable contribution to the research would be to explore the different effects of traits of in-group members and out-group members on leadership ostracism within an organization.

Third, in the organizational situation, case studies show that power distance, *Chaxu* climate, organizational culture, and the management system would reinforce or perpetuate the negative consequences of leadership ostracism. Situational factors influence management practice; for example, job-oriented and employee-oriented organizational cultures may lead to different leadership behavioral choices (Pheko et al., 2017). Therefore, organizational dynamics in how situational factors are recognized, function, and are applied can be further explored in the theoretical framework in future research.

Fourth, we applied the interview method using practical cases in an inductive manner to explore when and why supervisors engage in leadership ostracism and to establish a conceptual model that identifies the antecedent mechanism of leadership ostracism from supervisors, subordinates, and the organizational situation. Our model is consistent with the toxic triangle model of destructive leadership proposed by Padilla et al. (2007) and provides empirical testing of the following: (1) the narcissism and power consciousness of supervisors, (2) the self-concept of subordinates, and (3) the management system of the organizational situation.

Fifth, some distinctive features of leadership ostracism in the model are further found: specifically, stereotypes, and results orientation in the supervisor traits; political skills, job performance, and cognitive style in the subordinate traits; and power distance, *Chaxu* climate, organization politics in the organizational situation. This contributes to the research on leadership ostracism that distinguishes from destructive leadership and enriches the research on negative leadership. Additionally, we explored the specific influential factors in the Chinese culture, such as *Chaxu* climate (also referred to as *Chaxu geju*), which refers to a differential model based on different intimate relationships; however, this area of study merits further exploration that may elucidate triggers leading to leadership ostracism behaviors (Chen and Dian, 2018; Sun, 2019) and deepen our understanding of this social issue. This is especially significant in the Asian culture context.

Lastly, this paper evokes increased future investigation into the triadic interaction between supervisor, subordinate, and organizational situation. In the interactive model, one factor may increase or weaken the effects of another factor under certain circumstances (Mao et al., 2018). Moreover, similarities in the behaviors between supervisors and subordinates may trigger a sense of belonging and exert an interactive influence on leadership ostracism (Song and Kim, 2020).

### Practical Implications

Developing a conceptual integrated model is critical for managing the obscure and subtle destructiveness of leadership ostracism on employees and organizations (Akhtar et al., 2020). For supervisors, both strong supervisory skills and control (e.g., policies, command, or system) are the guarantee for maintaining effective management and achieving appropriate checks and balances in the workplace. When selecting or hiring for supervisory positions within an organization, human resource recruiters should pay particular attention to the leadership traits of the candidates, and those with destructive characteristics should be intentionally removed from consideration (Jahanzeb et al., 2018). For subordinates, increased training should be provided to guide employees to freely express themselves and to reject leadership ostracism. In the process, cultivating potential leaders among subordinates would enhance their cognitive and emotional traits when faced with ostracism (Xue et al., 2020). Regarding situational factors, the efforts to eliminate the influence of organizational culture, management system, power distance, and *Chaxu* climate may depend on organizational norms and values that indicate organizational resilience in the work process. This would include improvement measures in the areas of recruitment, training, job change, compensation and benefits, promotion, as well as other organizational systems (Pheko et al., 2017).

### Limitations and Future Studies

As with all studies, there are several limitations worth mentioning. First, the interview data are cross-sectional and primarily taken from subjective recollections of participants on distressing experiences whereby researchers cannot guarantee that variables affecting leadership ostracism should be covered as comprehensively as possible. Second, due to research materials, human resources, and financial and time constraints, the sample size is limited whereby further research on multinational cultures in other settings should be pursued. Third, this model focuses on individual interactive perspectives, with little attention on leadership ostracism toward a particular team/group target. Future research could explore the group-based leadership ostracism that is divided into intergroup ostracism and outgroup ostracism (Mao et al., 2018). Finally, the interactions between leadership, subordinates, and context in our model would benefit from further exploration. Results indicate that relatively independent factors in each domain constitute a simple additive model, and the more factors that occur simultaneously, the greater contribution of the domain to the emergence of leadership ostracism. For example, regarding subordinate characteristics, we see that the interaction between multiple factors such as social sensitivity, political skills, job performance, and self-efficacy is more likely to produce vulnerability in potential victims to leadership ostracism (Zhao et al., 2019). Furthermore, our research does not reveal whether these three influential domains (supervisor, subordinate, and context) produce a mutual offset or enhancement, thereby offering future researchers the opportunity to supplement our study with other empirical methods such as experiments and questionnaires.

## Data Availability Statement

The original contributions presented in the study are included in the article/supplementary material, further inquiries can be directed to the corresponding author/s.

## Ethics Statement

Ethical review and approval was not required for the study on human participants in accordance with the local legislation and institutional requirements. The patients/participants provided their written informed consent to participate in this study.

## Author Contributions

ZC: study design, evaluation, and revision. MS: get data and draft writing. All authors contributed to the article and approved the submitted version.

## Conflict of Interest

The authors declare that the research was conducted in the absence of any commercial or financial relationships that could be construed as a potential conflict of interest.

## Publisher's Note

All claims expressed in this article are solely those of the authors and do not necessarily represent those of their affiliated organizations, or those of the publisher, the editors and the reviewers. Any product that may be evaluated in this article, or claim that may be made by its manufacturer, is not guaranteed or endorsed by the publisher.

## Appendix A

**Interview protocol**

1. What is your direct supervisor like?
2. How do you get along with your supervisor?
3. Have you ever been ostracized, neglected and rejected by your supervisor?
4. Please describe a specific situation in which you are ostracized, neglected and rejected by your supervisor.
5. How do you respond to these unpleasant sufferings from your supervisor?
6. In your opinion, why does your supervisor ostracize, neglect or reject or neglect you? Please discuss it in terms of supervisor, subordinate and organizational environment factors.
7. Have any other colleagues in your organization ever been ostracized, neglected and rejected by the supervisor?
8. How do these colleagues get along with the supervisor?
9. Please describe a specific situation you have observed in which other colleagues have been ostracized, neglected and rejected by the supervisor.
10. How do your colleagues respond to these unpleasant sufferings from the supervisor?
11. In your opinion, why does the supervisor ostracize, neglect or reject these colleagues? Please discuss it in terms of supervisor, subordinate and organizational environment factors.
12. Supplementary contents.
